# Development of a hospice research culture through staff development, education and collaboration

**DOI:** 10.1136/bmjspcare-2018-001740

**Published:** 2019-04-16

**Authors:** Sarah Stanley, Alexandra McDougall, Laura Chapman, Amara Callistus Nwosu

**Affiliations:** 1 Marie Curie Hospice Liverpool, Liverpool, UK; 2 Aintree University Hospitals NHS Foundation Trust, Liverpool, UK; 3 Palliative Care Institute Liverpool, University of Liverpool, Liverpool, UK

**Keywords:** hospice, nursing care, palliative care, research, terminal care

Palliative care research is essential for evidence-based models of care that improve terminal illness outcomes.^1^ Investment in hospice research is essential for the projected increases in palliative needs.^2^ Hospices are crucial to practice and policy development, but there are many hospice research barriers.^3^ Well-documented problems include lack of research funding, governance challenges, methodological issues and absence of research culture.^1 4^ Hospices require strategic infrastructure and processes to progress from ‘research aware’ to full participation and research leadership.^3^ Over 2 years, Marie Curie Hospice Liverpool developed a series of quality improvement initiatives to establish a UK hospice research culture and improve research opportunities.

First, a governance process was established to facilitate the appropriate sponsorship and indemnity arrangements. This involved partnership with a local National Health Service Trust and University research teams. Patient and public engagement activities for research were established through our day therapy services. Patients met with researchers to provide perspectives for proposed research (from idea conception to research delivery). Second, a new morning board-round handover system provided focused clinical handover to hospice staff. As part of that, potential research participation eligibility was routinely discussed for all hospice inpatients. Third, Liverpool Clinical Commissioning Group funds provided a doctor (AMcD) and nurse (SS) with protected research time over 6 months to support National Institute for Health Research (NIHR) portfolio research studies. Initial duties involved training in hospice research processes. This included relevant training completion, data record management, ethical review facilitation and research promotion in the hospice. Our staff established contacts with the NIHR North West Clinical Research Network nurses in the local Trust. We focused on recruitment (patients and family caregivers) and data collection for the NIHR studies.

The process has led to several positive outcomes. Notably, patients were eager to participate in studies and showed great desire to improve future care, consistent with previous work.^5^ The process improved research opportunities for hospice patients, increased recruitment and created new research collaborations. Both staff members (AMcD and SS) developed research skill and knowledge, with one (SS) in receipt of a national award. Challenges encountered were time-related due to research governance process delays. Ongoing issues exist with funding for further work. We have recently appointed a research lead (ACN) to further support research delivery. In summary, the process demonstrated research improvement potential through steps to develop and engage hospice staff, secure funding and establish collaborative research partnerships. This activity has directly resulted in a growing research hospice culture and has potential as a model for other hospices.

## References

[1] Higginson IJ Research challenges in palliative and end of life care. BMJ Support Palliat Care 2016;6:2–4. 10.1136/bmjspcare-2015-001091 PMC4789687 26893386

[2] Etkind SN, Bone AE, Gomes B, et al How many people will need palliative care in 2040? past trends, future projections and implications for services. BMC Med 2017;15 10.1186/s12916-017-0860-2 PMC5436458 28514961

[3] Payne S, Preston N, Turner M, et al Research in palliative care - can hospices afford to not be involved? International Observatory on End of Life Care, University of Lancaster, 2013.

[4] Nwosu AC Integrated clinical academic training: an exciting new dawn for academic palliative medicine. J Palliat Med 2012;15:507–8. 10.1089/jpm.2011.0524 22577782

[5] Nwosu AC, Mayland CR, Mason S, et al Patients want to be involved in end-of-life care research. BMJ Support Palliat Care 2013;3:457 10.1136/bmjspcare-2013-000537 24950528

